# The Gambling Disorders Identification Test (GDIT): Psychometric Evaluation of a New Comprehensive Measure for Gambling Disorder and Problem Gambling

**DOI:** 10.1177/10731911211046045

**Published:** 2021-10-07

**Authors:** Olof Molander, Peter Wennberg, Anne H Berman

**Affiliations:** 1 Karolinska Institutet, Solna, Sweden; 2 Stockholm Region Health Services, Stockholm, Sweden; 3 Stockholm University, Stockholm, Sweden; 4 Uppsala University, Uppsala, Sweden

**Keywords:** gambling disorder identification test, GDIT, gambling disorder, problem gambling, at-risk gambling, psychometric evaluation

## Abstract

The novel gambling disorder identification test (GDIT) was recently developed in an international Delphi and consensus process. In this first psychometric evaluation, gamblers (*N* = 603) were recruited from treatment- and support-seeking contexts (*n* = 79 and *n* = 185), self-help groups (*n* = 47), and a population sample (*n* = 292). Participants completed self-report measures, a GDIT retest (*n* = 499), as well as diagnostic semistructured interviews assessing gambling disorder (GD; *n* = 203). The GDIT showed excellent internal consistency reliability (α = .94) and test–retest reliability (6-16 days, intraclass correlation coefficient = 0.93). Confirmatory factor analysis yielded factor loadings supporting the three proposed GDIT domains of gambling behavior, gambling symptoms, and negative consequences. Receiver operator curves and clinical significance indicators were used to estimate GDIT cut-off scores in relation to recreational (<15) and problem gambling (15-19), any GD (≥20), mild GD (20-24), moderate GD (25-29), and severe GD (≥30). The GDIT can be considered a valid and reliable measure to identify and predict GD severity, as well as problem gambling. In addition, the GDIT improves content validity in relation to an international research agreement concerning features of gambling outcome measures, known as the Banff Consensus Agreement.

Measuring gambling has long been a challenging issue within the gambling research field. While numerous instruments have been developed and validated with satisfactory psychometric properties (for reviews, see Caler et al., 2016; Pickering et al., 2017; Williams et al., 2012), most were developed before the introduction of the *Diagnostic and Statistical Manual of Mental Disorders–Fifth edition* (*DSM-5*), where the clinical criteria for gambling disorder (GD) were revised (American Psychiatric Association, 2013). In *DSM-5*, gambling was equated with alcohol and drugs and classified under substance-related and addictive disorders.

Furthermore, in similarity with alcohol use disorder and substance use disorder, diagnostic severity was introduced for GD within three levels. If 4 or 5 of 9 criteria are fulfilled, GD is labelled mild, 6 or 7 criteria yield a label of moderate GD, while 8 or 9 criteria yield a diagnosis of severe GD. A recent systematic review (Otto et al., 2020) concluded that current evidence for diagnostic accuracy is lacking, as only one of the gambling instruments identified, the *DSM-III*-based South Oaks Gambling Screen (SOGS; American Psychiatric Association, 1987; Goodie et al., 2013; Lesieur & Blume, 1987), had been validated in relation to *DSM-5* using semistructured diagnostic interviews. As such, how existing gambling measures relate to GD remains unclear, in particular in relation to levels of symptom severity.

## Measurement Issues in GD and Problem Gambling

Prevalence estimates of GD are scarce. Prevalence investigations have traditionally built on the concepts of “problem gambling” or “at-risk gambling,” two public health-based terms, where the former has been defined as “excessive gambling behavior that creates negative consequences for the gambler, others in his/her social network, and for the community” (Blaszczynski & Nower, 2002, p. 488), and the latter has been defined as “[gamblers] heavily involved in gambling, . . . [who] may or may not have experienced adverse consequences from gambling” (Ferris & Wynne, 2001). A systematic analysis found large variations in past-year prevalence for problem gambling worldwide, ranging from 0.12% to 5.8%; prevalence estimates from different studies were difficult to compare, as a variety of gambling instruments, cut-offs and time frames had been used to assess problem gambling (Calado & Griffiths, 2016). These measurement issues have been noted in additional reviews (see, e.g., Caler et al., 2016; Williams et al., 2012). In the same vein, gambling treatment has been characterized by a wide range of diverse outcome measures and domains assessing problem gambling (Toneatto & Ladouceur, 2003), causing difficulties when comparing effectiveness of different treatment approaches (Pallesen et al., 2005).

## The Banff Consensus Agreement (BCA)

To address these measurement issues, an expert panel of gambling researchers convened in 2004, at the Alberta Gambling Research Institute’s 3rd Annual Conference (Walker et al., 2006). A consensus-based framework known as the BCA, was formulated, specifying a set of minimal features of gambling outcome measures within three domains: (a) gambling behavior (net expenditures per month, frequency of gambling in days per month, and time spent thinking about gambling per month); (b) problems caused by gambling (mental health, relationships, financial, employment and productivity, and legal [a criterion later removed in the *DSM-5*; American Psychiatric Association, 2013]); and (c) treatment-specific measures of proposed mechanisms of change. Since then, the BCA has been influential as the basis for a proposed core set of reporting standards for gambling treatment studies, although it is unclear whether studies have actually adhered to these recommendations (Pickering et al., 2017), possibly due to construct underrepresentation (Spurgeon, 2017) in existing gambling measures.

## Content Validity of Frequently Used Gambling Measures in Relation to the BCA

While comprehensive reviews examining measurement content analysis in relation to the BCA seem to be lacking, both Pickering et al. (2017) and Molander et al. (2019) commented that most existing gambling measures appeared to fail to fulfill the recommendations of the BCA.

Using Domains 1 (gambling behavior) and 2 (problems caused by gambling) of the BCA as benchmarks, we analyzed the content of seven of the most frequently used gambling outcome measures identified in a systematic review (Pickering et al., 2017), as well as the frequently used public health measure problem gambling severity index (PGSI; Ferris & Wynne, 2001); see Table 1. Measures mostly included items for gambling-related financial, and relationship problems, but assessed health-related problems and gambling behavior more rarely. In addition, to fulfill the BCA recommendations on gambling behavior, it is necessary for item response alternatives to specify time units per month. Most measures, such as SOGS (Lesieur & Blume, 1987) and the NORC Diagnostic Screen for Gambling Problems (NODS; Wickwire et al., 2008), had dichotomous “Yes” or “No” responses or, such as in the PSGI (Ferris & Wynne, 2001), or verbal response alternatives, for example “not at all,” “rarely,” “sometimes,” or “often”; these are also aggravating factors for temporal ambiguity and poor item readability (i.e., difficulties in knowing how to interpret items; see Peter et al., 2018). In conclusion, none of the individual measures, nor any combinations of the measures analyzed seemed to sufficiently fulfill the features of BCA. Regarding Domain 3 of the BCA, this encompasses measures dependent on treatment-specific assumptions of therapeutic change, meaning that it was not feasible to capture all these possible theoretical constructs in a single instrument. This domain was therefore excluded from our analysis.

**Table 1.** Content Validity of Frequently Used Gambling Measures in Relation to the Recommended Features of the Banff Consensus Agreement.

Measures^a^	SOGS	*DSM-IV*	NODS	VGS	G-SAS	TLFB-G	PGSI
Gambling-related content of measure	Symptoms, *DSM-III* criteria	Symptoms, *DSM-IV* criteria	Symptoms, *DSM-IV* criteria	Harms, enjoyment	Urges, thoughts, and behaviors	Behaviors, time, and expenditures	Symptoms, *DSM-III* criteria
*Includes items assessing feature of the Banff consensus agreement*							
Gambling behavior per month							
Net expenditures	No	No	No	No	No	Yes	No
Days gambling	No	No	No	No	Partially^b^	Yes	No
Time preoccupation	No	No	No	No	Partially^c^	No	No
Problems caused by gambling							
Health	No	No	No	No	Yes	No	Yes
Relationships	Yes	Yes	Yes	Yes	Yes	No	No
Financial	Yes	Yes	Yes	Yes	Yes	No	Yes
Legal^d^	No	Yes	No	No	Yes	No	No

*Note*. SOGS = the South Oaks Gambling Screen (Lesieur & Blume, 1987); *DSM* = *Diagnostic and Statistical Manual of Mental Disorders; DSM-IV* = the criteria for pathological gambling in *DSM-IV* (American Psychiatric Association, 1994); NODS = the NORC Diagnostic Screen for Gambling Problems (Wickwire et al., 2008); VGS = the Victorian Gambling Screen (Tolchard & Battersby, 2010); G-SAS = the Gambling Symptom Assessment Scale (Suck Won Kim et al., 2009); TLFB-G = the timeline follow-back for gambling (Hodgins & Makarchuk, 2003; Weinstock et al., 2004); PGSI = the problem gambling severity index (Ferris & Wynne, 2001).

a All measures, except the PGSI, were used in 9% or more of the gambling studies identified in a systematic review by Pickering et al. (2017). ^b^Included, but not as days per month but rather hours during the past week. ^c^Included, but not per month but rather in the past week. ^d^The Banff consensus agreement was published before illegal acts to finance gambling were removed in the revised *DSM-5* criteria (American Psychiatric Association, 2013).

## Development of the Gambling Disorder Identification Test (GDIT)

As a response to these measurement challenges, a process was initiated to develop the Gambling Disorder Identification Test (GDIT), as a gambling measure analogous to the Alcohol Use Disorders Identification Test (AUDIT; Saunders et al., 1993), and the Drug Use Disorders Identification Test (DUDIT; Berman et al., 2005). The GDIT has been developed in several interdependent steps, using the BCA (Walker et al., 2006) as an overall benchmark. In the first step (Molander et al., 2019), four gambling researchers selected 30 items for proposed inclusion in GDIT, based on content analysis and categorization of a pool of 583 unique items from 47 existing gambling measures. In the second step, preliminary construct and face validity were established. Sixty-one gambling experts from 10 countries rated the 30 items proposed for inclusion in the GDIT, in an online Delphi process (see Molander et al., 2020), which is a systematic iterative method to determine expert consensus for research questions that are not suitable for experimental or epidemiological research designs, such as determining collective values or defining foundational concepts (Jorm, 2015; Yücel et al., 2019). Gambling researchers and clinicians participated in subsequent consensus meetings, yielding a 14-item draft version of GDIT, within three proposed domains. Gambling behavior (GDIT_Items 1-3_), corresponded to the features of gambling behavior in Domain 1 of the BCA. Gambling symptoms (GDIT_Items 4-10_), included key GD symptoms, which were not recommended in the BCA. Negative consequences (GDIT_Items 11-14._), corresponded to the features of problems caused by gambling, in Domain 2 of the BCA. In addition, an appendix to the GDIT assessed gambling expenditures and gambling types separately. Domain 3 of the BCA, measures of the processes of change, was not included in the GDIT, as these features could include a range of items assessing a multitude of treatment-specific assumptions and theoretical constructs. In similarity with the AUDIT (Saunders et al., 1993) and the DUDIT (Berman et al., 2005), the GDIT used frequency and time-based multiple choice response alternatives, which corresponded to the temporal features of the BCA. Face validity and item readability of the GDIT draft version were evaluated by obtaining user feedback from eight individuals with experience of problem gambling, as well as eight treatment-seeking participants fulfilling the criteria of GD (see Molander et al., 2020, for more details). In the final step, evaluation of the GDIT regarding psychometric properties was conducted among 603 gamblers from treatment- and support-seeking contexts, self-help groups, and population samples. The aim of this article is to report the results of this final step.

## Method

### Participants

Participants (*N* = 603) were recruited in four cohorts of gamblers. Recreational gamblers (*n* = 292), support-seeking gamblers (*n* = 185), participants in gambling self-help groups (*n* = 47), and treatment-seeking gamblers (*n* = 79), were recruited to an online survey via advertisements at self-help groups, at social media and online forums, and via clinicians at the Stockholm Center for Dependency Disorders. In the survey, participants reported if they had sought treatment for gambling within the health care system or social services the past year (treatment-seeking cohort), or visited a gambling self-help organization as a gambler the past year (self-help groups cohort). Participants who reported neither, but had gambled the past year, were defined as recreational gamblers. In addition, participants defined as support-seeking gamblers (*n* = 185) were recruited to a separate online survey, via advertisement at stodlinjen.se, the Swedish national gambling helpline. Recruitment began in November 2018 and ended in June 2020. Inclusion criteria were as follows: being ≥18 years old, and having gambled the past year, with the exception of the self-help group participants. All stages of the study were approved by the Regional Ethics Board of Stockholm, Sweden (ref. no. 2017/1479-31), and all participants provided informed consent for study participation and publication of results.

The mean age in the total sample was 33 years (*SD* = 12.1), with 74% men. The most predominant gambling type was playing casino online (51%). Table 2 shows further participant characteristics.

**Table 2.** Participant Characteristics by Cohort and Across Measure Points.

Measure point	1					2	3
Samples	Recreational (*n* = 292)	Support-seeking (*n* = 185)	Self-help groups (*n* = 47)	Treatment-seeking (*n* = 79)	Total (*N* = 603)	GDIT retest (*n* = 499)	Diagnostic interview (*n* = 203)
Demographic characteristics:							
Age *M* (*SD*)	29.5 (10.5)	35.2 (14)	40.1 (9.8)	36.6 (10)	33 (12.1)	32.8 (11.9)	37.3 (12)
Sex (%)							
Men	82	64	66	72	74	75	74
Women	17	36	34	24	25	24	26
Not stated	1	0	0	4	1	1	0
Source of income (%)							
Employed	60	65	68	77	64	63	67
Studies	30	15	11	5	21	22	13
Other^a^	10	20	21	18	15	15	20
Highest level of education (%)							
University	46	25	21	25	35	36	34
High school	45	56	68	62	52	52	53
Junior high school	8	15	9	10	10	10	9
Civil status (%)							
Cohabiting	54	49	64	49	52	53	55
Children	30	46	72	52	41	40	54
Gambling characteristics							
Gambling debts (%)	8	47	89	85	36	35	64
Gambling types (%)^b^							
Casino online	33	63	70	76	51	49	65
Casino land-based	12	11	15	13	12	12	15
Sport games online	53	31	38	43	44	44	38
Sport games venue	13	10	26	18	14	14	13
Poker online	20	13	13	18	17	17	15
Poker club	6	4	2	9	6	6	6
EGM	4	8	19	10	7	7	11
Number games	9	10	6	6	9	10	10
Lotteries	35	22	11	13	26	28	21
Horse betting	16	14	21	25	17	19	21
Bingo	9	11	15	9	10	11	8
Other	14	8	6	6	11	11	9

*Note*. GDIT = the gambling disorder identification test (Molander et al., 2019, 2020); EGM = electronic gambling machines.

a This category included unemployment insurance, income support, sickness compensation, sickness benefit, pension, and other sources of income. ^b^Participants were able to report several gambling types.

### Measures

An online survey was set up, including informed consent, demographic characteristics, and the following self-report questionnaires: GDIT (Molander et al., 2019, 2020), PGSI (Ferris & Wynne, 2001), the problem and pathological gambling measure (PPGM; Williams & Volberg, 2013), the NODS (Wickwire et al., 2008), the Adult ADHD Self-Report Scale (ASRS; Kessler et al., 2005), the Mood Disorder Questionnaire (MDQ; Hirschfeld et al., 2000), and the World Health Organization Quality of Life, 26-item version (WHOQOL-BREF; Skevington et al., 2004; see Table 3 for measure scores).

**Table 3.** Measure Scores by Cohort at Baseline.

Gambler cohorts	Recreational (*n* = 260-292)	Support-seeking (*n* = 144-185)	Self-help groups (*n* = 38-47)	Treatment-seeking (*n* = 57-79)	Total (*N* = 499-603)
	*M* (*SD*)	*M* (*SD*)	*M* (SD)	*M* (*SD*)	*M* (*SD*)
GDIT					
Total score	10.3 (9.6)	25.1 (16.1)	28.9 (16.6)	31.8 (14.8)	19.1 (15.8)
Gambling behavior	4.5 (3.0)	7.6 (4.5)	7 (6.6)	7.9 (6.1)	6.1 (4.6)
Gambling symptoms	4.0 (5.0)	11.8 (8.2)	13 (9.9)	14.5 (8.3)	8.5 (8.3)
Negative consequences	1.7 (2.9)	5.8 (5.3)	9.0 (4.1)	9.4 (3.8)	4.5 (5.0)
GDIT retest					
Total score	10 (9.2)	25.6 (16.4)	30.2 (16.4)	31.9 (15.5)	18.6 (15.8)
Gambling behavior	4.5 (3)	7.5 (4.6)	7.1 (6.6)	7.8 (6.3)	5.9 (4.5)
Gambling symptoms	3.9 (4.7)	11.9 (8.3)	13.9 (9.8)	14.8 (8.4)	8.2 (8.2)
Negative consequences	1.7 (2.9)	6.2 (5.2)	9.2 (4.1)	9.4 (3.8)	4.4 (5.0)
PGSI					
Total score	3.3 (5.2)	11.8 (9.0)	12.7 (9.9)	14.8 (8.1)	8.1 (8.8)
PPGM					
Total score	2.1 (3.2)	6.7 (4.9)	8.1 (5.3)	8.9 (4.7)	4.8 (5.0)
Problem	0.5 (1.2)	2.5 (2.3)	3.4 (2.6)	3.9 (2.1)	1.8 (2.3)
Impaired control	0.9 (1.3)	2.1 (1.4)	2.5 (1.6)	2.6 (1.4)	1.6 (1.6)
Other issues	0.5 (0.9)	1.6 (1.3)	1.6 (1.3)	1.9 (1.2)	1.1 (1.2)
ASRS					
Total score	26.9 (12.9)	28.7 (13.8)	32.8 (15.8)	30.5 (13.4)	28.4 (13.6)
MDQ					
Total score	4.2 (4.3)	5.3 (4.2)	6.9 (4.6)	6.9 (4.6)	5.1 (4.4)
WHOQOL-BREF					
Physical health	15.0 (2.9)	14.0 (2.9)	13.7 (3.3)	13.4 (3.3)	14.4 (3.1)
Psychological	13.6 (3.1)	12.3 (3.2)	11.9 (3.5)	12 (2.9)	12.9 (3.2)
Social relationships	14.3 (3.5)	13.3 (3.4)	12.9 (3.1)	12.8 (3.5)	13.7 (3.5)
Environment	15.1 (2.6)	13.9 (2.9)	13.8 (3.1)	13.3 (2.6)	14.4 (2.8)
SCI-GD, diagnostic interview	*n* = 43	*n* = 79	*n* = 31	*n* = 50	*n* = 203
No GD, *n* (%)	28 (65%)	39 (49%)	15 (48%)	12 (24%)	94 (17%)
Mild GD, *n* (%)	6 (14%)	16 (20%)	2 (6%)	10 (20%)	34 (17%)
Moderate GD, *n* (%)	9 (21%)	6 (8%)	8 (26%)	10 (20%)	33 (16%)
Severe GD, *n* (%)	—	18 (23%)	6 (19%)	18 (36%)	42 (21%)

*Note*. GDIT = the gambling disorder identification test (Molander et al., 2019, 2020); PGSI = the problem gambling severity index (Ferris & Wynne, 2001); PPGM = the problem and pathological gambling measure (Williams & Volberg, 2013); ASRS = the Adult Attention-Deficit/Hyperactivity Disorder Self-Reporting Rating Scale (Kessler et al., 2005); MDQ = the Mood Disorder Questionnaire (Hirschfeld et al., 2000); WHOQOL-BREF = the World Health Organization Quality of Life, 26-item version (Skevington et al., 2004); SCI-GD = the structured clinical interview for gambling disorder (Grant et al., 2004).

#### GDIT

The GDIT (Molander et al., 2019, 2020), previously described, is a newly developed gambling measure. The GDIT consists of 14 items with frequency-based response alternatives, in three domains: Gambling behavior GDIT_Items 1-3_ (scored 0-6), gambling symptoms GDIT_Items 4-10_ (scored 0-4), and negative consequences GDIT_Items 11-14_ (scored 0, 2 or 4). The maximum GDIT total score is 62. In addition, gambling expenditures and gambling types are assessed separately in an appendix to the GDIT. The GDIT is in the public domain, available at www.gditscale.com.

#### Structured Clinical Interview for Gambling Disorder (SCI-GD)

The SCI-GD (Grant et al., 2004) was used as a standard reference measure for GD. The SCI-GD is a clinician-administered semistructured interview assessing the *DSM-5* diagnostic criteria of GD, including levels of severity. If individuals meet 4-5 criteria GD is labeled mild, 5-6 criteria are labeled as moderate GD, and 7-9 criteria are labeled severe GD. The SCI-GD has shown excellent interrater and test–retest reliability, as well as high sensitivity (0.88) and specificity (1.00), based on longitudinal assessment in a gambling treatment-seeking sample (Grant et al., 2004).

### Design

**Figure 1.**
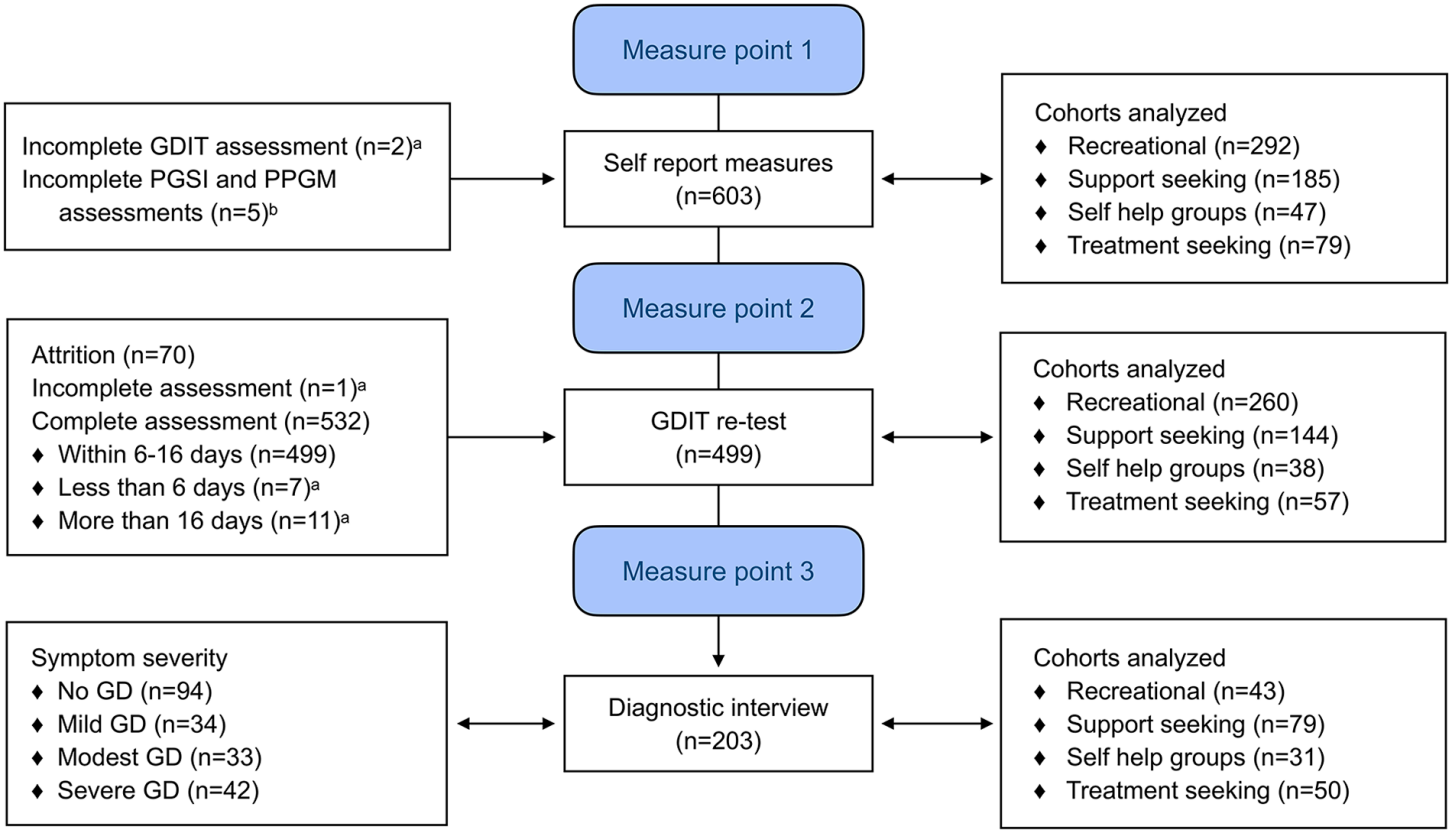
Study flow chart. *Note*. GDIT = the gambling disorder identification test (Molander et al., 2019, 2020); GD = gambling disorder; PGSI = the problem gambling severity index (Ferris & Wynne, 2001); PPGM = the problem and pathological gambling measure (Williams & Volberg, 2013). ^a^Removed from analysis. ^b^Removed from analyzes including PGSI and PPGM.

Participants completed informed consent, demographic characteristics, and self-report measures (GDIT; Molander et al., 2019, 2020; PGSI; Ferris & Wynne, 2001; PPGM; Williams & Volberg, 2013; NODS; Wickwire et al., 2008; ASRS; Kessler et al., 2005; MDQ; Hirschfeld et al., 2000; and WHOQOL-BREF; Skevington et al., 2004) in an initial online assessment. One week later, participants received an email with an invitation to complete a second online assessment for the GDIT retest. Finally, participants were invited to partake in a SCI-GD interview conducted by telephone, a reliable procedure for psychiatric assessment (Cantwell et al., 1997). Assessors were clinical psychologists and advanced clinical psychology students (*n* = 9), who prior to interviews had participated in a diagnostic workshop on GD and SCI-GD. Initially, all participants who completed the GDIT retest were contacted for an interview. Eventually, participants with higher gambling self-report scores were prioritized, to obtain at least 30 participants in every GD severity level. Each participant received two movie vouchers after completing the GDIT retest assessment (see Figure 1. for study flowchart).

### Statistical Analysis and Missing Data

A study protocol was published a priori (Molander et al., 2019), as well as an additional a priori statistical analysis plan (https://osf.io/6zpqs/). Two participants were excluded from the analysis, since they had missing data in the first GDIT assessment. Five participants had missing data for PGSI and PPGM, and were excluded from analyses including these measures. Furthermore, approximately 30% of participants had missing data for the NODS (Wickwire et al., 2008), due to a measurement error. Therefore, convergent validity was not estimated between GDIT and NODS, although this was originally part of the analysis plan. There were also additional small changes to the original analysis plan: (a) Aside from the confirmatory factor analysis using maximum likelihood, we also ran a model with the weighted least square mean and variance adjusted (WLSMV) estimator, to account for ordinal data. This model was included in view of the GDIT’s frequency and time-based multiple choice response alternatives. (b) We also tested for measurement invariance, as suggested by a reviewer. The SCI-GD was used as a reference standard for GD. PGSI and PPGM served as reference standards for at-risk and problem gambling, as well as for a clinical significance cut-off point (Jacobson & Truax, 1992) comparing recreational (norm population) with help-seeking samples (support seeking, self-help groups and treatment-seeking gamblers collapsed into one group). The clinical significance cut-off point *c* was estimated, as the populations were overlapping. All analyses were performed using R (version 4.1.0) and R Studio (1.4.1717) software (R Core Team, 2018), with the following key packages: psych, irr, cutpointr, lavaan, and semTools.

## Results

### Reliability

In the total sample, internal consistency reliability for the total GDIT score was excellent (α = .94) and good to excellent (α = .80-.94) for three domains of gambling behavior (GDIT_Items 1-3_), gambling symptoms (GDIT_Items 4-10_), and negative consequences (GDIT_Items 11-14._). Test–retest reliability, assessed at 6-16 days, was excellent for the total GDIT score (intraclass correlation coefficient [ICC] = 0.93) and good to excellent for the specific GDIT domains (ICC = 0.88-0.93). See Table 4 for estimates of test–retest reliability and internal consistency reliability in the specific cohorts. Cronbach alpha if item deleted was excellent for all GDIT items (raw α = .93-.94; standardized α = .93-.95), and corrected item-total correlations indicated very good discrimination for all GDIT items (.44-.89; see Table 4).

**Table 4.** Internal Consistency and Retest Reliability for the GDIT.

Gambler cohorts	Recreational	Support-seeking	Self-help groups	Treatment-seeking	Total
Cronbachs α^a^; *N* = 603	*n* =292	*n* = 185	*n* = 47	*n* = 79	*N* = 603
GDIT total score	0.90	0.93	0.91	0.89	0.94
Gambling behavior	0.73	0.82	0.94	0.89	0.85
Gambling symptoms	0.90	0.93	0.95	0.92	0.94
Negative consequences	0.65	0.78	0.71	0.51	0.80
Test–retest^b^ (6 to 16 days); *n* = 499	*n* = 260	*n* = 144	*n* = 38	*n* = 57	*N* = 499
GDIT total score	0.88	0.96	0.83	0.86	0.93
Gambling behavior	0.82	0.91	0.78	0.51	0.80
Gambling symptoms	0.87	0.91	0.66	0.90	0.90
Negative consequences	0.73	0.91	0.71	0.66	0.88

*Note*. GDIT = the gambling disorder identification test (Molander et al., 2019, 2020).

a Raw alpha. ^b^Intraclass correlation coefficient, two-way mixed effect model, and “single rater” unit with absolute agreement.

### Factor Structure

Confirmatory factor analysis was estimated testing a model of the three domains (GDIT_Items 1-3_, GDIT_Items 4-10_, and GDIT_Items 11-14_), using the WLSMV estimator. The model provided a good fit, χ^2^(74)= 176.436, *p* < .05; RMSEA (root mean square error of approximation) = 0.048; CFI (confirmatory fit index) = 0.975; TLI (Tucker–Lewis index) = 0.969 (Bowen & Guo, 2011). Factor loadings were excellent to very good (Comrey & Lee, 2016) for all items except for GDIT_Item 11_ and GDIT_Item 14_ (see Table 5). A similar model, using maximum likelihood, showed comparable item factor loadings (range 0.475-0.934), but slightly poorer goodness-of-fit indices, χ^2^(74) = 283.320, *p* < .05; RMSEA = 0.068; CFI = 0.967; TLI = 0.960.

**Table 5.** Corrected Item-Total Correlations and Confirmatory Factor Loadings for the GDIT (*N* = 603).

No.	Item	Corrected item-total correlation	Factor loadings in CFA^a^		
			Factor 1	Factor 2	Factor 3
1.	How often do you gamble?	0.62	0.74		
2.	How much time do you spend gambling on a typical day?	0.63	0.76		
3.	How much time do you spend thinking about gambling on a typical day?	0.78	0.94		
4.	How often have you tried to control, cut down or stop your gambling, in the past 12 months?	0.74		0.76	
5.	How often have you gambled to win back money you lost on gambling, in the past 12 months?	0.87		0.90	
6.	How often, in the past 12 months, have you gambled more than you planned (more occasions, longer time or larger sums)?	0.89		0.90	
7.	How often have you lied to others about your gambling, in the past 12 months?	0.84		0.86	
8.	How often have you borrowed money or sold something to obtain money for gambling, in the past 12 months?	0.80		0.82	
9.	How often have you gambled as a way of escaping problems or relieving negative feelings, in the past 12 months?	0.83		0.85	
10.	How often have you gambled with larger sums to get the same feeling of excitement as before, in the past 12 months?	0.80		0.82	
11.	Have you or anyone close to you experienced financial problems due to your gambling?	0.58			0.64
12.	Has your gambling worsened your mental health?	0.78			0.89
13.	Have you experienced serious problems in any important relationship because of your gambling?	0.70			0.78
14.	Have you experienced serious problems at work or in school because of your gambling?	0.44			0.48

*Note*. GDIT = the gambling disorder identification test (Molander et al., 2019, 2020); CFA = confirmatory factor analysis, using the weighted least square mean and variance adjusted estimator.

a Standardized factor loadings.

Measurement invariance was estimated using WLSMV. For men and women, configural and metric invariance showed good fit indices, χ^2^(148) = 232.137, *p* < .05; RMSEA = 0.044; CFI = 0.979; TLI = 0.975, and χ^2^(159) = 222.126, *p* < .05; RMSEA = 0.037; CFI = 0.984; TLI = 0.982, respectively, and did not differ significantly (Δχ^2^ =14.754, degrees of freedom [*df*] = 11, *p* = .194), indicating configural and metric invariance. The test of scalar invariance showed good fit indices, χ^2^(182) = 4240.759, *p* < .05; RMSEA = 0.037; CFI = 0.983; TLI = 0.982; and did not differ significantly from the metric model (Δχ^2^ =15.968, *df* = 10, *p* = .1006), when one item (GDIT_Item 14_) was released, which indicated partial scalar invariance for men and women. We also tested for measurement invariance regarding age groups (median split, 18-30 years vs. 31 and older). Configural and metric invariance showed good fit indices, χ^2^(148) = 242.778, *p* < .05; RMSEA = 0.046; CFI = 0.976; TLI = 0.970, and χ^2^(159) = 218.785, *p* < .05; RMSEA = 0.035; CFI = 0.985; TLI = 0.982, respectively, and did not differ significantly (Δχ^2^ =13.998, *df* = 11, *p* = .2331), indicating configural and metric invariance. Although the test of scalar invariance showed good fit indices, χ^2^(170) = 238.767, *p* < .05; RMSEA = 0.037; CFI = 0.982; TLI = 0.981, neither complete nor partial scalar invariance could be established. The difference between the scalar and metric model was significant (Δχ2 = 27.946, *df* = 11, *p* = .0033), and showed specific differences for most of the items (GDIT_Item 12_, GDIT_Item 13_, and GDIT_Item 14_) in the negative consequences domain. In conclusion, the measurement invariance analyses showed that the GDIT factor structure was mainly consistent across gender, and that the weakest item in terms of measurement invariance was GDIT_Item 14_. Regarding age, the differences in factor structure between young adults (18-30 years) and older participants, were attributable to items within the GDIT negative consequences domain.

### Convergent and Discriminant Validity

In the total sample, the GDIT total score showed positive correlations with the gambling measures PGSI and PPGM (*r* = .90 and *r* = .89, respectively), and with having gambling debts (*r* = .68), supporting convergent validity with these measures. Regarding discriminant validity, the GDIT total score showed much smaller positive correlations with measures assessing attention deficit hyperactivity disorder (ASRS; *r* = .37) and bipolar disorder (MDQ; *r* = .34), as well as negative correlations with various domains related to quality of life (WHOQOL-BREF; *r* = −.40 to −.30). See Table 6 for estimates regarding the specific cohorts.

**Table 6.** Convergent and Divergent Validity of the GDIT.

Gambler cohorts (*n* = 598)	Recreational	Support-seeking	Self-help groups	Treatment-seeking	Total
Convergent validity					
PGSI					
GDIT total score	0.87	0.90	0.77	0.81	0.90
Gambling behavior	0.59	0.68	0.39	0.62	0.65
Gambling symptoms	0.86	0.89	0.83	0.76	0.89
Negative consequences	0.75	0.78	0.49	0.51	0.78
PPGM					
GDIT total score	0.83	0.90	0.77	0.81	0.89
Gambling behavior	0.60	0.73	0.42	0.57	0.66
Gambling symptoms	0.83	0.86	0.81	0.79	0.88
Negative consequences	0.68	0.78	0.50	0.54	0.78
Divergent validity					
ASRS					
GDIT total score	0.27	0.43	0.39	0.43	0.37
Gambling behavior	0.13	0.35	0.19	0.39	0.27
Gambling symptoms	0.29	0.42	0.35	0.30	0.35
Negative consequences	0.26	0.39	0.42	0.39	0.34
MDQ					
GDIT total score	0.23	0.38	0.10	0.27	0.34
Gambling behavior	0.10	0.31	0.13	0.13	0.22
Gambling symptoms	0.25	0.32	0.06	0.23	0.31
Negative consequences	0.24	0.39	0.04	0.36	0.37
WHOQOL-BREF^a^					
Physical health	–0.24	–0.36	–0.31	–0.19	–0.34
Psychological	–0.29	–0.54	–0.29	–0.32	–0.43
Social relationships	–0.29	–0.32	–0.12	–0.12	–0.30
Environment	–0.34	–0.39	–0.36	–0.09	–0.40

*Note*. ASRS = the Adult ADHD Self-Report Scale (Kessler et al., 2005); GDIT = the gambling disorder identification test (Molander et al., 2019, 2020); MDQ = the Mood Disorder Questionnaire (Hirschfeld et al., 2000); PGSI = the problem gambling severity index (Ferris & Wynne, 2001); PPGM = the problem and pathological gambling measure (Williams & Volberg, 2013); WHOQOL-BREF = the World Health Organization Quality of Life, 26-item version (Skevington et al., 2004).

a Estimates for convergent and divergent validity were calculated in relation to the GDIT total score, as well as the GDIT domains gambling behavior, gambling symptoms, and negative consequences; except for the WHOQOL-BREF domains who were estimated only in relation to the GDIT total score.

### Diagnostic Accuracy

Receiver operator curves (ROCs) were primarily estimated for the GDIT total score in relation to GD severity levels (no GD, mild, moderate, and severe), assessed by SCI-GD, and approximate cut-off score ranges were selected based on Youden’s index, generally prioritizing sensitivity over specificity. The results (see online supplementary Table 1) indicated that a GDIT total score between 20 and 24 had a sensitivity of 0.84 to 0.89, a specificity of 0.74 to 0.79, and Youden’s index of 0.60 to 0.65, with an area under the curve [AUC] of 0.88, corresponding to mild GD; a GDIT total score of 25 to 29 (sensitivity = 0.83-0.84, specificity = 0.66-0.84, Youden’s index = 0.53-0.57, AUC = 0.84) corresponded to moderate GD, and a GDIT total score of 30 or more (sensitivity = 0.95, specificity = 0.68, Youden’s index = 0.64, AUC = 0.86) corresponded to severe GD. A GDIT total score of ≥20 corresponded to any GD level.

As a complementary analysis, GDIT total score ROCs were estimated in relation to at-risk and problem gambling assessed by PGSI and PPGM, as well as the clinical significance cut-off point *c* (Jacobson & Truax, 1992). A GDIT total score of between 10 and 14 corresponded to at-risk gambling cut-off scores according to the PGSI and PPGM (sensitivity = 0.70-0.85, specificity = 0.86-0.95, Youden’s index = 0.66-0.71, AUC = 0.93). A GDIT total score of between 15 and 19 corresponded to problem gambling cut-off scores according to the PGSI and PPGM (sensitivity = 0.86-0.96, specificity = 0.87-0.95, Youden’s index = 0.81-0.85, AUC = 0.97; see online supplementary Table 2), as well as to the clinical significance cut-off point *c* (*c* = 16.7; see Table 7, for a summary of GDIT cut-off scores). Finally, PGSI and PPGM scores (means and standard deviations), were estimated for the identified GDIT cut-off score ranges (see Table 8).

**Table 7.** GDIT Cut-Off Scores.

	Recreational gambling^a^	Problem gambling^a^	Gambling disorder^b^			
			Any	Mild	Moderate	Severe
GDIT total score (range 0-62)	<15	15-19	≥20	20-24	25-29	≥30

*Note*. GDIT = the gambling disorder identification test (Molander et al., 2019, 2020).

a Estimated in relation to the problem gambling severity index (PGSI; Ferris & Wynne, 2001) and the problem and pathological gambling measure (PPGM; Williams & Volberg, 2013), as well as the clinical significance cut-off point *c* (Jacobson & Truax, 1992). ^b^Estimated in relation to the structured clinical interview for gambling disorder (SCI-GD; Grant et al., 2004).

**Table 8.** GDIT Cut-Off Score Ranges in Relation to PGSI and PPGM (*n* = 598).

GDIT total	PGSI total	PPGM total
Score ranges	*M* (*SD*)	*M* (*SD*)
At-risk gambling 10-14	3.11 (3.15)	2.30 (2.27)
Problem gambling 15-19	6.27 (3.76)	4.71 (3.38)
Mild gambling disorder 20-24	9.43 (4.91)	6.78 (2.72)
Modest gambling disorder 25-29	13.16 (5.65)	8.68 (3.17)
Severe gambling disorder ≥30	19.22 (4.70)	10.81 (2.72)

*Note*. GDIT = the gambling disorder identification test (Molander et al., 2019, 2020); PGSI = the problem gambling severity index (Ferris & Wynne, 2001); PPGM = the problem and pathological gambling measure (Williams & Volberg, 2013).

## Discussion

This study evaluated the psychometric properties of a novel gambling measure, the GDIT. The GDIT was developed in a recent international Delphi and consensus process, aiming to establish a comprehensive measure which corresponded to a previous international research agreement regarding features of gambling outcome measures, known as the BCA. A further aim was to develop a measure that could identify and assess fulfilment of the revised diagnostic criteria for GD, for example, levels of symptom severity.

GDIT total scores increased continuously when comparing samples with increasing levels of presumed symptom severity, that is, recreational gamblers, support-seeking gamblers, gamblers in self-help groups, and treatment-seeking gamblers. The GDIT showed estimates of convergent and discriminant validity that corresponded to theoretical expectations, and excellent estimates on most reliability statistics. Factor loadings based on confirmatory factor analysis were excellent to very good with the exception of one item, and supported the three proposed theoretical domains: gambling behavior (GDIT_Items 1-3_), gambling symptoms (GDIT_Items 4-10_), and negative consequences (GDIT_Items 11-14_). Finally, ROC and clinical significance cut-off estimates yielded approximate GDIT cut-off score ranges for assessment of gambling severity. Overall, we conclude that GDIT can be viewed as a valid and reliable measure to identify and predict severity of GD, as well as problem gambling.

Regarding item inclusion, some psychometric estimates indicated possible construct irrelevance (Spurgeon, 2017), suggesting that the GDIT could be shortened. The GDIT_Item 14_, measuring school or work-related problems due to gambling, showed lower, but still acceptable, reliability estimates (i.e., corrected item-total correlations and factor loadings), in comparison with other GDIT items The GDIT_Item 14_ was also problematic in terms of measurement invariance for men and women, specifically scalar invariance. For young adults (18-30 years) and older, the analyses of measurement invariance showed differences for most of the items (GDIT_Item 12_, GDIT_Item 13_, and GDIT_Item 14_) within the negative consequences domain. The finding of invariance for negative consequences was not surprising, as gambling-related negative consequences can be expected to affect younger and older individuals differently due to varying life circumstances. Also, several GDIT items had high corrected item-total correlations.

Although these findings all indicate that GDIT items could to some extent be eliminated, we are mindful that the BCA (Walker et al., 2006) recommended that the features covered by these items should be included in gambling measures; furthermore, the aforementioned items were also rated in the Delphi expert process as important to include in the GDIT (Molander et al., 2020). Therefore, we decided to retain the GDIT item structure that resulted from the consensus meetings, prioritizing content validity over performance. Still the lower performance of GDIT_Item 14_, in relation to the other GDIT items is noteworthy. In the GDIT item selection (Molander et al., 2019), it became evident that many items from previous gambling instruments assessed gambling-related school, work, and relationships problems lumped together in single items, equivalent to current and previous *DSM* diagnostic criteria (American Psychiatric Association, 1994, 2013). This particular double- and triple-barreled phrasing issue was also observed in the Delphi process (Molander et al., 2020) and was later adjusted, during the consensus meetings, into two GDIT items, assessing relationship problems in one item (GDIT_Item 13_), and gambling-related school or work problems in another (GDIT_Item 14_). Although the BCA (Walker et al., 2006) recommends that gambling-related problems concerning employment and productivity should be measured as a single feature in gambling instruments, most psychometric studies have evaluated gambling-related problems regarding work and/or relationships combined (i.e., in single items using double-barreled phrasing). The lower performance of GDIT_Item 14_, compared with GDIT_Item 13_, might indicate that gambling-related work problems (quite apart from relationship problems), might be less relevant than previously assumed. Anecdotally, several participants expressed during the SCI-GD interviews that their gambling actually made them more efficient at work rather than causing problems, as working was their primary source of obtaining money to continue gambling.

While showing promising psychometric properties, the largest contribution of the GDIT to the gambling field will probably lie in content analysis from a theoretical point of view. The GDIT closes a gap with the inclusion of several constructs that have been recommended in previous consensus-based agreements among gambling researchers (Walker et al., 2006), but have been lacking in existing gambling instruments. Furthermore, the GDIT was developed analogously to the AUDIT (Saunders et al., 1993) and the DUDIT (Berman et al., 2005), in line with the *DSM-5* decision to equate gambling with alcohol and substance use, all as addictive disorders (American Psychiatric Association, 2013). The GDIT includes frequency- and time-based assessments, which is an advantage compared with most existing gambling instruments. For example, the gambling behavior domain (GDIT_Items 1-3_), assesses gambling behavior in frequency of events and duration of hours, using response scales similar to the AUDIT (Saunders et al., 1993) and the DUDIT (Berman et al., 2005). This enables future research to examine specific time-related cut-offs for gambling behavior in relation to gambling-related constructs, as well as to facilitate comparisons between gambling behavior and substance use. Furthermore, although outside the scope of this evaluation, GDIT encompasses additional assessment of gambling expenditures in relation to income, as well as differentiation between gambling types at online versus physical venues.

This study had several strengths. We included gamblers from several populations, enabling us to present sample-specific estimates within a broad range of gambling contexts. The psychometric evaluation was preceded by several documented and interdependent development steps, including results from an international Delphi process with a large proportion of currently active gambling researchers participating, and consensus-based agreements among gambling researchers (Molander et al., 2019, 2020). While this is the first study evaluating psychometric properties of GDIT, it is also one of the first psychometric studies estimating diagnostic accuracy of a gambling measure in relation to semistructured diagnostic interviews assessing GD, also considering levels of severity. This is important, as a recent systematic review of gambling screening instruments (Otto et al., 2020), concluded that there is a lack of evidence for GD diagnostic accuracy. This study showed different GDIT cut-off scores for each GD severity level, which is important from dual perspectives of clinical utility and public health. For example, it is now possible to concurrently conduct prevalence estimates of diagnosed GD in different populations, in addition to assessing the prevalence of problem or at-risk gambling.

The study also had some limitations. One limitation was that GDIT estimates of prevalence accuracy for at-risk and problem gambling were more uncertain than the estimates in relation to GD, as they did not rely on comparisons with semistructured interviews, but rather on other self-report instruments: that is, PGSI and PPGM. As a complementary analysis, we estimated clinical significance (Jacobson & Truax, 1992), which indicated that problem gambling, but not at-risk gambling, corresponded to the cut-off differentiating between recreational and help-seeking gambling populations. Overall, at-risk and problem gambling are more loosely defined terms than GD, and it is less clear how to validly establish cut-off scores for these constructs. A second possible limitation was that there were small differences in demographic characteristics between the total sample, and the subsamples that complemented the GDIT retest and diagnostic interviews. An exception was that the proportion of participants with gambling debts was higher in the subsample that completed the diagnostic interviews compared with the total sample (64% vs. 36%), but smaller compared with the treatment-seeking cohort (64% vs. 85%). The higher proportion of gambling debts among those who completed a diagnostic interview might indicate a higher gambling severity. This is not necessarily a limitation of the study, as the primary purpose of the diagnostic interview was to assess a sufficient subsample of participant within the full GD severity spectrum (i.e., no GD, mild GD, moderate GD, and severe GD), to be able to estimate diagnostic accuracy of the GDIT. A third limitation is the low overall internal consistency for the negative consequences scale, as well as the low test–retest estimates for the gambling behavior subscale among treatment-seeking gamblers. The lower internal consistency for the negative consequences domain, among the recreational and treatment-seeking gamblers, may have to do with Cronbach α being contingent on item number, where the negative consequences domain is only four items. This emphasized the need to use the GDIT as a full instrument, as opposed to using separate subscales for each domain. Fourth, test–retest estimates for gambling behavior were lower among treatment-seeking gamblers, compared with the total score and the other domains. This is not necessarily a limitation, but rather may reflect the inclusion in this domain of items assessing behaviors that capture short-term changes, which may be typical of treatment-seeking gamblers. The test–retest analysis thus includes a measure both of reliability, but also of actual behavior change, specific to the treatment-seeking group. A final limitation of the study was that assessment of gambling-related expenditures in the GDIT appendix was not validated; this assessment was included in the GDIT as a recommended feature from the BCA.

Future research should thus include validation of GDIT assessment of expenditures and corroboration of GDIT severity score ranges. In addition, future psychometric evaluation of GDIT could include examination of item structure using item response theory (Reise & Waller, 2009), or validation through comparisons of GDIT scores between different gambling types in physical or digital milieus. Of special interest are also international comparisons among different gambling samples.

## Supplemental Material

sj-pdf-1-asm-10.1177_10731911211046045 – Supplemental material for The Gambling Disorders Identification Test (GDIT): Psychometric Evaluation of a New Comprehensive Measure for Gambling Disorder and Problem Gambling Click here for additional data file. Supplemental material, sj-pdf-1-asm-10.1177_10731911211046045 for The Gambling Disorders Identification Test (GDIT): Psychometric Evaluation of a New Comprehensive Measure for Gambling Disorder and Problem Gambling by Olof Molander, Peter Wennberg and Anne H Berman in Assessment

sj-pdf-2-asm-10.1177_10731911211046045 – Supplemental material for The Gambling Disorders Identification Test (GDIT): Psychometric Evaluation of a New Comprehensive Measure for Gambling Disorder and Problem Gambling Click here for additional data file. Supplemental material, sj-pdf-2-asm-10.1177_10731911211046045 for The Gambling Disorders Identification Test (GDIT): Psychometric Evaluation of a New Comprehensive Measure for Gambling Disorder and Problem Gambling by Olof Molander, Peter Wennberg and Anne H Berman in Assessment

sj-pdf-3-asm-10.1177_10731911211046045 – Supplemental material for The Gambling Disorders Identification Test (GDIT): Psychometric Evaluation of a New Comprehensive Measure for Gambling Disorder and Problem Gambling Click here for additional data file. Supplemental material, sj-pdf-3-asm-10.1177_10731911211046045 for The Gambling Disorders Identification Test (GDIT): Psychometric Evaluation of a New Comprehensive Measure for Gambling Disorder and Problem Gambling by Olof Molander, Peter Wennberg and Anne H Berman in Assessment
