# Supplementary material for: The Gambling Disorders Identification Test (GDIT): Psychometric Evaluation of a New Comprehensive Measure for Gambling Disorder and Problem Gambling
Source: Assessment. 2021 Oct 7;30(1):225–37. doi: 10.1177/10731911211046045 (PMC9684656; doi:10.1177/10731911211046045)
Supplement: sj-pdf-1-asm-10.1177_10731911211046045 – Supplemental material for The Gambling Disorders Identification Test (GDIT): Psychometric Evaluation of a New Comprehensive Measure for Gambling Disorder and Problem Gambling [file sj-pdf-1-asm-10.1177_10731911211046045.pdf]

GDIT Gambling Disorder Identification Test

Here are a few questions on gambling. We thank you for responding as correctly and honestly as possible by indicating the response that is right for you.

☐ Man ☐ Woman ☐ Other alternative Age ______

1. How often do you gamble?	Never ☐	Monthly or less ☐	2-4 times a month ☐	2-3 times a week ☐	4 or more times a week ☐	Daily ☐	Several times a day ☐
2. How much time do you spend gambling on a typical day?	No time ☐	Less than an hour ☐	1-2 hours ☐	3-4 hours ☐	5 -6 hours ☐	7-9 hours ☐	10-24 hours ☐
3. How much time do you spend thinking about gambling on a typical day?	No time ☐	Less than an hour ☐	1-2 hours ☐	3-4 hours ☐	5 -6 hours ☐	7-9 hours ☐	10-24 hours ☐

4. How often have you tried to control, cut down or stop your gambling, in the past 12 months?	Never ☐	Less often than monthly ☐	Monthly ☐	Weekly ☐	Daily or almost daily ☐
5. How often have you gambled to win back money you lost on gambling, in the past 12 months?	Never ☐	Less often than monthly ☐	Monthly ☐	Weekly ☐	Daily or almost daily ☐
6. How often, in the past 12 months, have you gambled more than you planned (more occasions, longer time or larger sums)?	Never ☐	Less often than monthly ☐	Monthly ☐	Weekly ☐	Daily or almost daily ☐
7. How often have you lied to others about your gambling, in the past 12 months?	Never ☐	Less often than monthly ☐	Monthly ☐	Weekly ☐	Daily or almost daily ☐
8. How often have you borrowed money or sold something to obtain money for gambling, in the past 12 months?	Never ☐	Less often than monthly ☐	Monthly ☐	Weekly ☐	Daily or almost daily ☐
9. How often have you gambled as a way of escaping problems or relieving negative feelings, in the past 12 months?	Never ☐	Less often than monthly ☐	Monthly ☐	Weekly ☐	Daily or almost daily ☐
10. How often have you gambled with larger sums to get the same feeling of excitement as before, in the past 12 months?	Never ☐	Less often than monthly ☐	Monthly ☐	Weekly ☐	Daily or almost daily ☐

[PROOFREAD VERSION]

11. Have you or anyone close to you experienced financial problems due to your gambling?	No ☐	Yes, but not in the past year ☐	Yes, in the past year ☐
12. Has your gambling worsened your mental health?	No ☐	Yes, but not in the past year ☐	Yes, in the past year ☐
13. Have you experienced serious problems in any important relationship because of your gambling?	No ☐	Yes, but not in the past year ☐	Yes, in the past year ☐
14. Have you experienced serious problems at work or in school because of your gambling?	No ☐	Yes, but not in the past year ☐	Yes, in the past year ☐

Turn page

© 2020 Olof Molander, Rachel Volberg, Per Binde, Kristina Sundqvist, Peter Wennberg, Viktor Månsson & Anne H Berman.
English DRAFT version. Karolinska Institutet, Stockholm, Sweden. Correspondence: olof.molander@ki.se.
