# Supplementary material for: The Gambling Disorders Identification Test (GDIT): Psychometric Evaluation of a New Comprehensive Measure for Gambling Disorder and Problem Gambling
Source: Assessment. 2021 Oct 7;30(1):225–37. doi: 10.1177/10731911211046045 (PMC9684656; doi:10.1177/10731911211046045)
Supplement: sj-pdf-2-asm-10.1177_10731911211046045 – Supplemental material for The Gambling Disorders Identification Test (GDIT): Psychometric Evaluation of a New Comprehensive Measure for Gambling Disorder and Problem Gambling [file sj-pdf-2-asm-10.1177_10731911211046045.pdf]

Reviewer(s)' Comments to Author:

Reviewer: 1

Comments to the Author
The authors report a psychometric evaluation of the GDIT using a large sample of individuals who gamble at various levels of involvement, from recreational to treatment seeking. They utilize both self-report and interview-based measures to estimate indices of both reliability and validity. The GDIT has an exceptional measurement development history, and overall their results are compelling. Most of my remarks can be considered minor, and generally commenting on places where the writing could be improved for the sake of clarity. More substantially, the manuscript would be strengthened by adding measurement invariance testing, given their attention to factor structure, and additional information regarding how their total sample differed from the sample used to test diagnostic accuracy (i.e., the ones who completed the diagnostic interview). Overall, strong paper.

Specific comments are provided below, grouped by major paper sections.

Abstract:
1. Authors use the term 'Banff' in the abstract, which is likely to be unfamiliar to a large portion of the readers. Consider removing this sentence or altering it such that this general point can be made but without using this unfamiliar term. Another option would be to briefly define Banff for the readers.
Answer: Thank you for this valuable comment. We have revised the abstract (page 3, line 55), and also used the term Banff Consensus Agreement (BCA) consistently throughout the manuscript.

Introduction:
2. Introduction is very well written. It highlights the most pressing concerns regarding measurement issues in the detection of gambling disorder. One omission is a discussion of the poor readability of the most widely used gambling disorder measures. Consider commenting on this. This could be mentioned during the discussion of temporal ambiguity in response options (page 6), which is one component of readability/difficulty knowing how to interpret items. Citation for your consideration below, where the issue of vagueness/ambiguity of questions on gambling disorder questionnaires is discussed:
Peter, S. C., Whelan, J. P., Pfund, R. A., & Meyers, A. W. (2018). A text comprehension approach to questionnaire readability: An example using gambling disorder measures. Psychological Assessment, 30(12), 1567-1580.
Answer: Thank you for this excellent comment, and for a very interesting reference. We have commented on temporal ambiguity and poor readability, accordingly (see page 6, line 128).

3. It was a bit confusing seeing the three proposed domains of the GDIT (behavior, symptoms, consequences) in the abstract and then seeing the intro focus on the three components of the Banff consensus (behavior, problems caused by gambling, mechanisms of change). As I reader, I found myself trying to figure out if these three were supposed to correspond to each other in some way. I think if the authors found a way to address this explicitly, it would help the overall clarity of the intro.
Answer: Thank you for pointing this out. We agree that this section of the introduction was less clear, and thank you for the opportunity to clarify this. We have revised the manuscript, accordingly (page 7, line 157).

4. Excellent argument for current gambling measures underrepresenting constructs from the Banff consensus. Very much appreciated this claim being substantiated in the content validity section of the intro.
Answer: Thank you very much, we appreciate that you acknowledged this. Content validity has indeed been an important part of the GDIT development process.

5. A brief definition of a "Delphi process" is warranted.

Answer: Thank you for this excellent suggestion, which we agree on. We have refined the Delphi method in the manuscript accordingly (page 7, line 152).

6. Some description of "user feedback" would be helpful. What were the individuals experiencing problem gambling or treatment-seeking individuals asked to comment on? Item clarity? Validity? Also, any sort of description of whether their feedback resulted in changes to the items would be helpful. If that is detailed in another article, a reference to that would be appropriate.
Answer: Thank you for these questions. We used a "think aloud" procedure, where the participants were instructed to comment freely after reading instructions and items, e.g., on item structure, response alternatives or phrasing. The user feedback led to a revision of the GDIT draft version. However, as you guessed, this process is described in detail in a previous article (Molander et al., 2020). Thus, we followed your suggestion and added a reference to this in the manuscript (page 8, line 178).

Method:

7. Some description of the difference between "support seeking" versus "treatment seeking" would be helpful, especially since the authors highlight this spectrum at the start of the discussion section. Perhaps this was defined by recruitment method, or how they described themselves? More information needed to understand how these are meaningfully different.
Answer: Thank you for this suggestion. We have clarified the recruitment methods and differences between the support-seeking and treatment seeking cohorts (page 8, line 187).

8. For participant characteristics, Table 2, it would be helpful to see more complete breakdowns of levels of the variables. For example, you note the recreational sample was 82% men. This implies 18% "other than men". Was there a response option for non-binary gender identities (e.g., transgender)? Similar with other categories – 60% employed implies 40% "other than employed." Of course, this will make the table a bit denser, but it would be helpful for other authors, especially those wanting to do reviews of representation in studies such as yours. It may be relevant for the poor performance of the work/school-related item as well, which I remark on in the discussion section of this review.
Answer: Thank you for this excellent suggestion. We have revised Table 2 accordingly, also adding characteristics of the sub-samples that completed the re-test assessment and diagnostic interviews (page 27, line 625).

9. Any significant differences between those participants who self-selected to complete the diagnostic interview and those who did not? This would be worthwhile to note given how much smaller the sample that completed the diagnostic interview was compared to the starting sample. Any implications that would follow if it is a significantly different demographic sample should be noted in the limitations of the manuscript.
Answer: Thank you for these important queries. When revising demographic characteristics in Table 2, we noticed few differences between the total sample, and the sub-sample that completed the interviews. We thus did not feel that it was necessary to do any formal significance testing regarding differences. An exception though, was that the proportion of participants with gambling debts was much higher in the sub-sample that completed the diagnostic interviews compared to the total sample (64% vs 36%), but smaller than in the treatment-seeking sample (85%). We have commented on this in the limitation section in the discussion (page 17, line 458).

10. The convergent/divergent (perhaps more appropriately referred to as convergent/discriminant) validity correlations all seem to make sense and is a nice feature of the manuscript. Still, I think it'd be more accurate to describe a correlation of .3-.4 as moderately strong than weak. I maintain that these correlations make sense, given the empirical overlap between ADHD and mood disorders with gambling disorder, and the relation between gambling and QOL. So it appears this is performing as expected from both a convergent and discriminant standpoint, and this can be maintained while still appropriately describing the magnitude of the correlations.

Answer: Thank you for these remarks. We have replaced "divergent validity" with "discriminant validity" throughout the manuscript. We agree that the interpretations of the convergent/discriminant correlations are not accurate, but preferred to describe the correlations in more relative terms, in the revised manuscript (page 12, line 295).

11. Regarding diagnostic accuracy, the authors provide estimates for detecting mild, moderate, and severe GD. Could they also provide an estimate for detecting GD at any level? In other words, what is the ideal cut-off score if one had to differentiate simply the presence of gambling disorder or not? Perhaps they did provide this and I am simply misunderstanding their description, in which case some added clarity would be helpful.
Answer: Thank you for this important point. Actually, the GDIT cut-off score for Any GD level is based on the score for Mild GD (the lower threshold), since the ROC Mild GD estimation compares a cut-off between No GD, versus Mild GD, Modest GD and Severe GD. Still, it is a very important comment, we agree that readers would want to know the estimate for Any GD level. We have revised the manuscript accordingly (page 13, line 313 and 320).

12. Regarding factor structure, the paper would benefit from at least some amount of measurement invariance testing. Does the GDIT perform similarly across gender identities and/or age categories? Another option would be to report invariance testing across recreational and non-recreational gamblers. One would hope that the GDIT, designed to assess gambling across involvement levels would perform equally well across these levels. Demonstrating this would greatly strengthen the paper. If the factor structure is not invariant across categories, this should be noted as a limitation of the measure, and something to consider if a shortened version of this measure was developed, like with the AUDIT-C.
Answer: Thank you for this suggestion. As a crude indicator of measurement invariance, we tested whether the GDIT performed equally well across gender, age and recruitment groups in terms of fit indices and factor loadings. See page 12, line 284, in the manuscript.

Discussion:

13. Page 13, sentence that begins, "This seems to be a result of our procedure in the preparatory development study for the GDIT…" is very difficult to comprehend. Overall, even after several re-reads, I had a difficult time understanding the point(s) that was being made in this paragraph. My understanding is that although item 14 was an outlier regarding performance, it was retained for theoretical reasons. I think some revision of this paragraph, with an emphasis of simplification/readability, would be helpful.
Answer: Thank you for this important comment, which we agree on. We have tried to clarify this section of the discussion (page 15, line 364).

14. Related to item 14, which asks about problems gambling has caused in work and/or school, is it possible this item performed poorly because only 60% of the sample was described as being "employed." Perhaps nearly half of this sample was not in school or working. This could merely be noted as a possible reason for the poor performance of that item.
Answer: Thank you for an interesting point. In the total sample, 64% were employed, 21% were studying and 15% had other sources of income, such as unemployment insurance, sickness compensation or pension (see the revised table 2). So, 85% of the sample were working or studying. The poor performance of item 14 might have other explanations; we have revised the Discussion regarding these, as also suggested by reviewer 2 (page 15, line 369).

15. For research and clinical purposes, I imagine the most common application of the GDIT will be to use the total score to differentiate individuals with and without gambling disorder, or perhaps "recreational" from "other" in a clinical context specifically. Some explicit identification/reiteration of that type single cut off score would be helpful.
Answer: Thank you for an important comment, which we agree on. To clarify the GDIT cut-off scores, we have revised the abstract (page 3, line 51), and also added an additional table in the manuscript summarizing GDIT cut-off scores for recreational gamblers, problem gamblers, and the severity levels for

gambling disorder (see Table 7, page 34, line 670).

Reviewer: 2

Comments to the Author
The authors present a study on the psychometric properties of the Gambling Disorder Identification Test (GDIT), proposed as a valid and reliable measure to identify gambling disorder according to the DSM-5 criteria, analogously to the AUDIT and the DUDIT. The paper is generally well written but, in my opinion, there are some major and minor points that need to be addressed in order to improve the manuscript. After these changes, it will be possible to judge whether the research is suitable for publication in Assessment.

Major revisions:
1. I would suggest the authors to stress the novelty of the proposed measure of GD in the Abstract.
Answer: Thank you for this excellent suggestion. We have revised the abstract accordingly (page 3, line 42).

2. In the Introduction, the authors said "where the clinical criteria for gambling disorder (GD) were revised (American Psychiatric Association, 2013)". I would recommend authors to specify the changes introduced by the DSM-5 and to better specify why the existing measures are limited.
Answer: Thank you for an important suggestion, which we agree on. We have clarified this section in the Introduction (page 3, line 42)

3. The response options for each item are not clear. I therefore recommend that the authors report, in another table or in appendix, the whole test, including instructions and scoring procedure.
Answer: Thank you for this comment. We prefer to distribute the GDIT (including future translations) via an external webpage https://gditscale.com/. Currently the English version of the GDIT is in proofread version (the psychometric evaluation was conducted using the Swedish GDIT version). Still, we acknowledge the need for reviewers to see the instrument. As a suggestion, we submit the English proofread version as editorial material, not to be published.

4. At page 4, the authors stated "Prevalence estimates of GD are scarce. Prevalence investigations have traditionally built on the concepts of "problem gambling" or "at-risk gambling", two public health-based terms". In my opinion, the proposed instrument for gambling assessment is also a measure of problem gambling given that the GD diagnosis is possible only with diagnostic tools or clinical interview. The GDIT, such as AUDIT and DUDIT, is a screening tool.
Answer: Thank you for this interesting comment. Our primary aim was to establish cut-off scores for the GDIT in relation to the severity levels of Gambling Disorder (GD), to be able to use GDIT as a screener to identify and assess GD (in the same vein as e.g., AUDIT can be used to assess Alcohol Use Disorder). As a complementary analysis, we also estimated GDIT cut-offs for problem gambling. We have clarified this in the Discussion (page 14, line 337) and Results (page 13, line 312 and 322).

5. At page 9, the authors stated "Participants completed informed consent, demographic characteristics and self-report measures in an initial online assessment". I would recommend the authors to specify, in brackets, the acronyms of the administered measure, for greater clarity.
Answer: Thank you for this suggestion. We have revised the manuscript accordingly (page 10, line 240).

6. In the Figure 1, the authors reported that 7 participants completed the second online assessment less than 6 days later, whereas 11 participants more than 16 days later. Were these 18 participants excluded from the analyses?
Answer: Thank you. Yes, these participants were removed/excluded from the analysis. An [a] was missing, marking a note in Figure 1 clarifying this. We have revised Figure 1 accordingly (see page 42, line 708).

7. How the authors explain the low internal consistency (.65 and .51) of negative consequences scale and the low re-test reliability of gambling behavior subscale among treatment-seeking gamblers?
Answer: Thank you for an interesting comment. Regarding the low internal consistency of the negative consequences among recreational and treatment seeking gamblers, we are not that surprised. The Cronbach alpha is contingent on the number of items, and the negative consequences subscale is only four items. The GDIT is expected to be used as a full scale, rather than single subscales. Regarding gambling behavior, this subscale includes items assessing behaviors that capture short term changes. Consequently, the test-retest analysis include both reliability and actual behavior change. We addressed both these issues in the limitation section of the Discussion (page 16, line 469).

8. The factor loading of the scale "negative consequences" are not very good. The authors should address this "problem".
Answer: Thank you. We agree that one item, item 14, has a rather low factor loading. However, items 11-13 had factor loadings between 0.66 to 0.87, which we consider satisfactory. Concerning item 14, which reviewer 1 also commented on, we have revised and extended the Discussion on why we finally decided to keep it, even though the psychometric properties call for caution (page 15, line 361).

9. In my opinion, the authors should estimate one-factor model of the GDIT, along with the three-factor model (that the authors have already reported in the manuscript).
Answer: Thank you for this suggestion. From a theoretical point of view, we assumed that there are three underlying factors in the GDIT. We tested a one-factor solution, and it showed a worse fit to data (RMSEA=0.128, CFI=0.882 , TLI=0.860). We have chosen to retain the three-factor solution in the manuscript.

10. The authors should consider that the factor loadings of item 11 and 14 are not very good.
Answer: Thank you. See the answer to comment 8 above.

11. I would recommend beginning the discussion with a brief introduction in which the authors specify the need of a new screening tool for gambling severity.
Answer: Thank you for this suggestion. We have revised the Discussion, accordingly (page 14, line 337).

12. At page 12, the authors stated "Factor loadings based on confirmatory factor analysis were excellent to very good with the exception of one item". I think that, along with item 14, the item 11 should be included.
Answer: Thank you. Good suggestion. We have revised the manuscript, accordingly (page 12, line 284).

Minor revisions:
1. At page 9, the authors stated "See Table 3 for estimates of test-retest reliability and internal consistency reliability in the specific cohorts" but the table with these values is the Table 4.
Answer: Thank you. We have revised the manuscript accordingly (page 11, line 273).

2. The reference to Table 3, in the text of the manuscript, is missing.
Answer: Thank you. We have revised the manuscript accordingly (page 9, line 217).
