# Supplementary material for: The Gambling Disorders Identification Test (GDIT): Psychometric Evaluation of a New Comprehensive Measure for Gambling Disorder and Problem Gambling
Source: Assessment. 2021 Oct 7;30(1):225–37. doi: 10.1177/10731911211046045 (PMC9684656; doi:10.1177/10731911211046045)
Supplement: sj-pdf-3-asm-10.1177_10731911211046045 – Supplemental material for The Gambling Disorders Identification Test (GDIT): Psychometric Evaluation of a New Comprehensive Measure for Gambling Disorder and Problem Gambling [file sj-pdf-3-asm-10.1177_10731911211046045.pdf]

GDIT total cut-off scores in relation to severity levels of Gambling Disorder (n=203)

GDIT scores	Mild GD (AUC=0.88)			Modest GD (AUC=0.84)			Severe GD (AUC=0.86)		
	SE	SP	YI	SE	SP	YI	SE	SP	YI
0	1.00	0.00	0.00	1.00	0.00	0.00	1.00	0.00	0.00
2	1.00	0.01	0.01	1.00	0.01	0.01	1.00	0.01	0.01
3	1.00	0.06	0.06	1.00	0.05	0.05	1.00	0.04	0.04
4	1.00	0.13	0.13	1.00	0.09	0.09	1.00	0.07	0.07
5	1.00	0.24	0.24	1.00	0.18	0.18	1.00	0.14	0.14
6	1.00	0.27	0.27	1.00	0.20	0.20	1.00	0.16	0.16
7	0.99	0.33	0.32	0.99	0.24	0.23	1.00	0.20	0.20
8	0.99	0.36	0.35	0.99	0.27	0.25	1.00	0.22	0.22
9	0.99	0.38	0.37	0.99	0.28	0.27	1.00	0.23	0.23
10	0.98	0.44	0.42	0.97	0.32	0.29	1.00	0.27	0.27
11	0.97	0.47	0.44	0.96	0.34	0.30	1.00	0.29	0.29
12	0.96	0.52	0.48	0.96	0.39	0.35	1.00	0.33	0.33
13	0.96	0.54	0.51	0.96	0.41	0.37	1.00	0.34	0.34
14	0.96	0.56	0.53	0.96	0.42	0.38	1.00	0.35	0.35
15	0.95	0.59	0.54	0.95	0.44	0.38	1.00	0.37	0.37
16	0.94	0.61	0.55	0.95	0.46	0.41	1.00	0.39	0.39
17	0.94	0.67	0.61	0.95	0.52	0.46	1.00	0.43	0.43
18	0.93	0.69	0.62	0.93	0.53	0.46	1.00	0.45	0.45
19	0.90	0.70	0.60	0.93	0.56	0.50	1.00	0.48	0.48
20	0.89	0.71	0.60	0.93	0.58	0.51	1.00	0.49	0.49
21	0.89	0.74	0.63	0.93	0.60	0.53	1.00	0.51	0.51
22	0.88	0.76	0.64	0.92	0.61	0.53	1.00	0.52	0.52
23	0.86	0.79	0.65	0.89	0.63	0.53	1.00	0.55	0.55
24	0.84	0.79	0.63	0.88	0.64	0.52	0.98	0.56	0.54
25	0.83	0.81	0.64	0.88	0.66	0.54	0.98	0.58	0.55
26	0.81	0.84	0.65	0.84	0.69	0.53	0.95	0.61	0.56

27	0.79	0.85	0.64	0.84	0.71	0.55	0.95	0.63	0.58
28	0.77	0.85	0.62	0.83	0.72	0.55	0.95	0.64	0.59
29	0.75	0.86	0.61	0.83	0.74	0.57	0.95	0.66	0.61
30	0.72	0.87	0.60	0.80	0.76	0.56	0.95	0.68	0.64
31	0.67	0.87	0.54	0.73	0.77	0.50	0.86	0.70	0.55
32	0.65	0.87	0.52	0.72	0.77	0.49	0.83	0.70	0.54
33	0.63	0.87	0.51	0.71	0.78	0.49	0.81	0.71	0.52
34	0.61	0.87	0.48	0.69	0.80	0.49	0.79	0.72	0.51
35	0.58	0.89	0.47	0.67	0.82	0.49	0.79	0.75	0.54
36	0.53	0.9	0.44	0.61	0.84	0.45	0.79	0.79	0.57
37	0.50	0.9	0.4	0.57	0.84	0.42	0.74	0.80	0.54
38	0.47	0.91	0.38	0.56	0.87	0.43	0.71	0.82	0.53
39	0.41	0.95	0.36	0.49	0.90	0.39	0.60	0.84	0.44
40	0.38	0.97	0.34	0.47	0.93	0.40	0.60	0.88	0.48
41	0.33	0.97	0.30	0.41	0.94	0.35	0.52	0.89	0.42
42	0.32	0.97	0.29	0.40	0.94	0.34	0.50	0.89	0.39
43	0.31	0.97	0.28	0.39	0.94	0.32	0.48	0.89	0.37
44	0.28	0.97	0.24	0.35	0.95	0.29	0.40	0.90	0.31
45	0.25	0.98	0.23	0.32	0.96	0.28	0.36	0.91	0.27
46	0.22	0.98	0.20	0.29	0.97	0.26	0.33	0.93	0.26
47	0.20	0.98	0.18	0.28	0.98	0.26	0.33	0.94	0.27
48	0.18	0.98	0.16	0.25	0.98	0.23	0.31	0.94	0.25
49	0.17	0.99	0.16	0.24	0.98	0.22	0.31	0.96	0.27
50	0.15	0.99	0.14	0.20	0.98	0.18	0.29	0.97	0.25
51	0.14	0.99	0.13	0.20	0.99	0.19	0.29	0.98	0.26
52	0.12	0.99	0.11	0.17	0.99	0.17	0.24	0.98	0.21
53	0.09	0.99	0.08	0.13	0.99	0.13	0.21	0.99	0.20
54	0.08	0.99	0.07	0.12	0.99	0.11	0.19	0.99	0.18
55	0.06	0.99	0.05	0.09	0.99	0.09	0.17	0.99	0.16

56	0.06	0.99	0.04	0.08	0.99	0.07	0.14	0.99	0.14
57	0.04	1.00	0.04	0.05	1.00	0.05	0.10	1.00	0.10
59	0.02	1.00	0.02	0.03	1.00	0.03	0.05	1.00	0.05

GDIT = The Gambling Disorder Identification Test (Molander et al., 2019, 2020)

GD = Gambling Disorder

AUC = Area under the curve

SE = Sensitivity

SP = Specificity

YI = Youdens index

Supplementary table 2

GDIT total cut-off scores in relation to at-risk and problem gambling (n=598)

GDIT scores	PGSI (AUC=0.93)			PPGM (AUC=0.93)			PGSI (AUC=0.97)			PPGM (AUC=0.93)		
	SE	SP	YI	SE	SP	YI	SE	SP	YI	SE	SE	YI
0	1.00	0.00	0.00	1.00	0.00	0.00	1.00	0.00	0.00	1.00	0.00	0.00
1	1.00	0.03	0.03	1.00	0.02	0.02	1.00	0.01	0.01	1.00	0.02	0.02
2	1.00	0.06	0.06	0.99	0.05	0.04	1.00	0.03	0.03	1.00	0.04	0.04
3	0.99	0.26	0.25	0.99	0.23	0.22	1.00	0.15	0.15	1.00	0.16	0.16
4	0.98	0.42	0.40	0.98	0.38	0.36	1.00	0.25	0.25	1.00	0.27	0.27
5	0.95	0.57	0.53	0.96	0.53	0.50	1.00	0.36	0.36	0.99	0.38	0.37
6	0.94	0.66	0.6	0.95	0.62	0.57	1.00	0.43	0.43	0.99	0.45	0.44
7	0.91	0.77	0.68	0.92	0.71	0.62	0.98	0.51	0.50	0.99	0.54	0.53
8	0.89	0.82	0.71	0.90	0.76	0.66	0.98	0.57	0.55	0.98	0.59	0.58
9	0.85	0.88	0.73	0.87	0.84	0.71	0.98	0.65	0.63	0.98	0.68	0.66
10	0.82	0.89	0.71	0.85	0.86	0.71	0.98	0.69	0.67	0.98	0.72	0.71
11	0.79	0.92	0.70	0.82	0.89	0.70	0.97	0.73	0.70	0.98	0.77	0.74
12	0.75	0.94	0.69	0.77	0.91	0.68	0.97	0.79	0.76	0.95	0.80	0.75
13	0.73	0.95	0.68	0.76	0.94	0.71	0.96	0.82	0.78	0.95	0.84	0.79
14	0.70	0.95	0.66	0.74	0.95	0.70	0.96	0.84	0.81	0.95	0.86	0.81
15	0.68	0.96	0.64	0.72	0.96	0.67	0.96	0.87	0.83	0.93	0.89	0.81
16	0.66	0.97	0.63	0.70	0.96	0.66	0.95	0.89	0.85	0.91	0.90	0.81
17	0.63	0.98	0.61	0.67	0.98	0.65	0.93	0.92	0.85	0.89	0.92	0.82
18	0.62	0.98	0.59	0.65	0.98	0.63	0.92	0.93	0.85	0.88	0.93	0.81
19	0.60	0.98	0.57	0.64	0.99	0.62	0.91	0.94	0.85	0.86	0.95	0.81
20	0.59	0.98	0.56	0.63	0.99	0.62	0.90	0.95	0.86	0.85	0.95	0.80
21	0.56	0.99	0.55	0.59	1.00	0.59	0.87	0.97	0.83	0.82	0.96	0.78
22	0.55	0.99	0.54	0.58	1.00	0.58	0.86	0.97	0.82	0.80	0.96	0.76
23	0.53	0.99	0.52	0.56	1.00	0.56	0.83	0.97	0.80	0.77	0.96	0.73
24	0.52	0.99	0.51	0.55	1.00	0.55	0.82	0.97	0.79	0.76	0.96	0.73

25	0.50	0.99	0.50	0.53	1.00	0.53	0.80	0.98	0.78	0.74	0.97	0.72
26	0.48	0.99	0.48	0.51	1.00	0.51	0.78	0.99	0.77	0.71	0.98	0.69
27	0.47	0.99	0.46	0.49	1.00	0.49	0.75	0.99	0.74	0.69	0.98	0.67
28	0.46	0.99	0.45	0.48	1.00	0.48	0.74	0.99	0.73	0.68	0.98	0.67
29	0.44	0.99	0.44	0.47	1.00	0.47	0.72	0.99	0.71	0.67	0.98	0.65
30	0.43	1.00	0.43	0.45	1.00	0.45	0.70	1.00	0.69	0.65	0.99	0.63
31	0.40	1.00	0.40	0.43	1.00	0.43	0.66	1.00	0.65	0.62	0.99	0.61
32	0.38	1.00	0.38	0.41	1.00	0.41	0.62	1.00	0.62	0.58	0.99	0.58
33	0.36	1.00	0.36	0.39	1.00	0.39	0.59	1.00	0.59	0.55	0.99	0.54
34	0.35	1.00	0.35	0.37	1.00	0.37	0.56	1.00	0.56	0.53	1.00	0.53
35	0.32	1.00	0.32	0.34	1.00	0.34	0.52	1.00	0.51	0.49	1.00	0.48
36	0.29	1.00	0.29	0.31	1.00	0.31	0.47	1.00	0.47	0.45	1.00	0.44
37	0.27	1.00	0.27	0.28	1.00	0.28	0.43	1.00	0.43	0.41	1.00	0.41
38	0.25	1.00	0.25	0.27	1.00	0.27	0.41	1.00	0.41	0.39	1.00	0.39
39	0.22	1.00	0.22	0.23	1.00	0.23	0.36	1.00	0.35	0.34	1.00	0.34
40	0.20	1.00	0.20	0.22	1.00	0.22	0.33	1.00	0.33	0.31	1.00	0.31
41	0.18	1.00	0.18	0.19	1.00	0.19	0.29	1.00	0.29	0.27	1.00	0.27
42	0.16	1.00	0.16	0.17	1.00	0.17	0.26	1.00	0.26	0.25	1.00	0.25
43	0.15	1.00	0.15	0.16	1.00	0.16	0.25	1.00	0.25	0.23	1.00	0.23
44	0.13	1.00	0.13	0.14	1.00	0.14	0.21	1.00	0.21	0.20	1.00	0.20
45	0.12	1.00	0.12	0.12	1.00	0.12	0.19	1.00	0.19	0.18	1.00	0.18
46	0.11	1.00	0.11	0.11	1.00	0.11	0.18	1.00	0.18	0.17	1.00	0.17
47	0.10	1.00	0.10	0.10	1.00	0.10	0.16	1.00	0.16	0.15	1.00	0.15
48	0.09	1.00	0.09	0.09	1.00	0.09	0.14	1.00	0.14	0.14	1.00	0.14
49	0.08	1.00	0.08	0.08	1.00	0.08	0.12	1.00	0.12	0.12	1.00	0.12
50	0.06	1.00	0.06	0.06	1.00	0.06	0.10	1.00	0.10	0.09	1.00	0.09
51	0.06	1.00	0.06	0.06	1.00	0.06	0.09	1.00	0.09	0.09	1.00	0.09
52	0.05	1.00	0.05	0.06	1.00	0.06	0.09	1.00	0.09	0.08	1.00	0.08
53	0.04	1.00	0.04	0.04	1.00	0.04	0.07	1.00	0.07	0.06	1.00	0.06

54	0.03	1.00	0.03	0.04	1.00	0.04	0.06	1.00	0.06	0.05	1.00	0.05
55	0.03	1.00	0.03	0.03	1.00	0.03	0.04	1.00	0.04	0.04	1.00	0.04
56	0.02	1.00	0.02	0.03	1.00	0.03	0.04	1.00	0.04	0.04	1.00	0.04
57	0.02	1.00	0.02	0.02	1.00	0.02	0.03	1.00	0.03	0.03	1.00	0.03
59	0.01	1.00	0.01	0.01	1.00	0.01	0.02	1.00	0.02	0.02	1.00	0.02
61	0.00	1.00	0.00	0.01	1.00	0.01	0.01	1.00	0.01	0.01	1.00	0.01
62	0.00	1.00	0.00	0.00	1.00	0.00	0.00	1.00	0.00	0.00	1.00	0.00

GDIT = The Gambling Disorder Identification Test (Molander et al., 2019, 2020)

GD = Gambling Disorder

AUC = Area under the curve

SE = Sensitivity

SP = Specificity

YI = Youdens index
